# From prompts to genomes: A constraint-first framework for AI-driven sequence design across life and viruses

**DOI:** 10.1371/journal.pcbi.1014865

**Published:** 2026-10-15

**Authors:** Adhityo Wicaksono, Arli Aditya Parikesit

**Affiliations:** 1 Aether Biomics, Tanah Abang, Jakarta, Indonesia; 2 Department of Biotechnology, School of Health and Life Sciences, i3L University, Pulo Gadung, Jakarta, Indonesia; Yale School of Public Health, UNITED STATES OF AMERICA

## Abstract

A constraint-first framework for future ‘prompt-to-genome’ AI across Bacteria, Archaea, Eukaryota, and viruses is proposed. Genome-scale models can now generate long DNA sequences, and experimentally viable genome-scale designs have begun to appear, but sequence generation alone does not establish biological feasibility. Constraints should not require designs to remain within natural sequence space; rather, they should define testable boundaries for function, inheritance, lifecycle coherence and safe use, including new-to-nature designs. We propose a two-part system. First, a Constraint Compiler translates natural-language prompts into machine-checkable specifications defining scope, context, objectives, assumptions and governance requirements. Second, a modular Validator Stack (C1–C6) evaluates candidate designs for sequence integrity, gene and regulatory plausibility, genome completeness, comparative coherence, systems-level feasibility, and safety and governance. Extreme prompts serve as stress tests to expose hidden assumptions, conflicting objectives, and refusal points, complemented by adversarial testing and risk assessment. A phased roadmap and community benchmarks are proposed to measure specification accuracy, biological feasibility, robustness, and governance performance. The goal is to move prompt-to-genome systems from impressive generation toward reliable, auditable, and accountable biological design.

## 1. The new bottleneck: Feasibility, not fluency

Recent genome-scale AI models have moved from long-sequence generation toward experimentally testable design. Evo and Evo 2 demonstrate genomic modeling and generation from molecular to genome scale [1,2], while genome language models have now produced viable bacteriophages with substantial evolutionary novelty [3]. These advances make ‘prompt-to-genome’ workflows increasingly plausible, but they do not make feasibility equivalent to sequence fluency. Genomes still depend on coordinated replication, inheritance, regulation, epigenetic state, and lifecycle requirements. Additionally, viral genomes depend on packaging and host context [4–9]. Biological constraints should not be interpreted as requiring generated sequences to remain within naturally observed sequence space. A central promise of generative biology is new-to-nature design: sequences may diverge substantially from known homologues while remaining functional [3]. The relevant boundary is therefore not naturalness itself, but compatibility with physical, molecular, hereditary, and system-level requirements of the intended function. The same novelty can also increase uncertainty because similarity-based evidence becomes weaker, strengthening the need for functional validation and safety assessment.

An important distinction is therefore needed between sequence-conditioned generation and specification-conditioned design. Current genome language models primarily generate candidates from nucleotide context and learned sequence distributions rather than directly translating arbitrary natural-language requirements into compliant genomes. In the recent bacteriophage demonstration, genome models supplied candidate sequences, while external constraints on sequence quality, coding content, host tropism, genetic architecture, and evolutionary diversity were used to select designs for experimental testing [3]. Thus, demonstrated capability resides in the complete design pipeline (i.e., generator, filtering, ranking, and validation) rather than in the generator alone. A future prompt-to-genome system would additionally require biological requirements expressed in natural language to become explicit, jointly satisfiable constraints before generation.

Extreme prompts expose additional scale-dependent constraints. In eukaryotes, for example, a ‘bigger genome’ does not imply more genes: genome expansion often reflects repeats and transposable elements [10–13]. A generator that lengthens a plant genome without accounting for gene structure, repeats, or inheritance could therefore produce plausible-looking but biologically incoherent sequences. In bacteria, feasibility includes replicon organization and segregation [4,14]. In eukaryotes, feasibility additionally depends on chromosome architecture and extensive noncoding sequence, while in viruses, packaging, lifecycle, and host compatibility [5–7]. Prompt-to-genome should therefore be treated as a hierarchy of design tasks rather than a single endpoint:

1. Gene-level: Coding and regulatory elements and noncoding RNA (including isoforms)
2. Module: Operons, pathways, cassettes, and viral segments
3. Replicon: Chromosome, replicon with architecture, and inheritance constraints
4. Whole genomes: Complete cellular or viral genome

Feasibility requirements increase with scale. Underspecified prompts should be converted into explicit assumptions before generation, and outputs should be returned as a constrained design space, validator-linked feasibility report, or auditable refusal (Fig 1).

**Fig 1:**
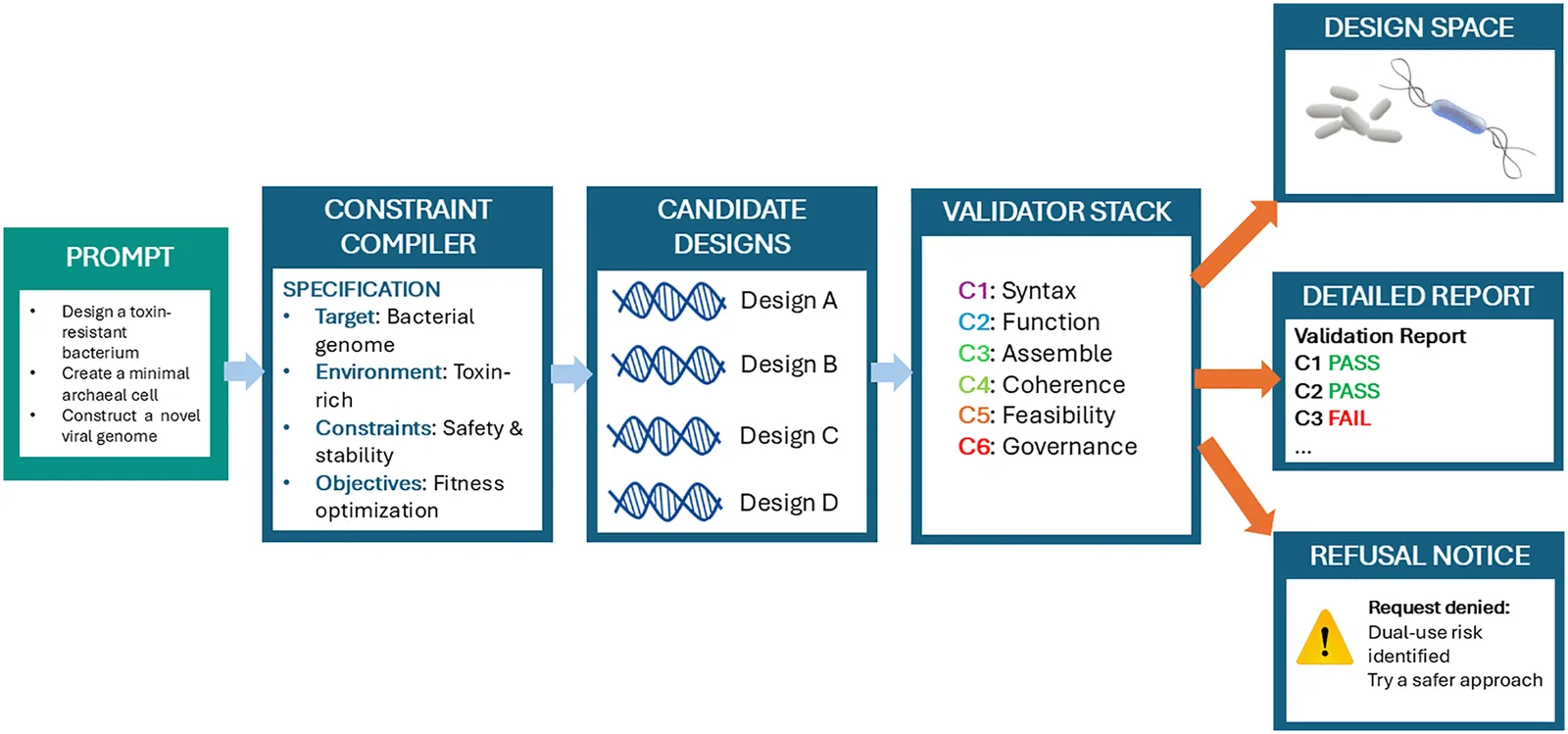
Constraint-first prompt-to-genome workflow across life and viruses. Prompts are compiled to specifications, candidates are evaluated by a modular Validator Stack (C1-C6), and outputs are a constrained design space, a detailed validation report, or a refusal notice.

## 2. A Constraint Compiler: Turning prompts into executable biological specifications

Simple ‘prompt-to-sequence’ systems usually leave key requirements implicit. A Constraint Compiler should translate prompts into a formal, machine-checkable specification before sequence generation. Existing design languages and standards such as Eugene, SBOL, Cello and CELLM show how biological intent can be represented as enforceable rules [15–18]. Proto now extends this direction into composable generative workflows across DNA, RNA, proteins and other modalities, incorporating predictive models, multi-objective design and natural-language specification through AI agents [19]. Proto therefore represents an emerging step toward specification-conditioned design because it separates sequences, generators, constraints and optimizers while allowing complex design programs to be specified through natural-language interfaces. Proto is also complementary to the framework proposed here: it addresses how generative design programs can be composed and optimized, whereas the Constraint Compiler defines an auditable biological-and-governance contract that downstream validators must test. At a narrower scale, DNA-Diffusion demonstrates conditional generation of regulatory DNA followed by computational and experimental activity testing [20], illustrating how generator-specific objectives can sit beneath a broader validation architecture. The compiled specification should define target scope (i.e., gene, module, replicon or whole genome), measurable functional goals, hard constraints, soft optimization objectives, assumptions about environment and containment, and governance fields such as intended use, synthesis-screening requirements and refusal triggers. Missing context should trigger clarification rather than silent inference.

Large language models can assist with interpreting user intent, but formal tools should encode and verify the resulting contract [17–19]. Feasibility should be treated as a multi-layer test suite rather than a single score. This mirrors genome assessment, where no single metric is sufficient: QUAST provides assembly diagnostics [21], BUSCO estimates conserved gene completeness [22], CheckM evaluates microbial completeness and contamination [23], Prokka standardizes prokaryotic annotation [24], and OrthoFinder supports comparative orthology analysis [25]. These tools were not designed as generative-AI validators but illustrate how complementary diagnostics can be composed. The proposed stack, whose implementation will differ by biological domain and design scale, is:

1. C1: Sequence integrity (alphabet, forbidden motifs, assembly and syntax).
2. C2: Gene and regulatory plausibility (ORFs, splicing, regulatory grammar and viral organization).
3. C3: Genome and content completeness (required components; BUSCO- or CheckM-style tests).
4. C4: Comparative and annotation coherence (annotation, orthology and architecture).
5. C5: Systems-level feasibility (physiology, lifecycle and FBA/COBRA-style tests where applicable) [26–28].
6. C6: Safety, biosecurity and governance (risk tiering, synthesis screening and auditable refusal).

Relying on a single evaluation score can encourage proxy optimization and obscure failures elsewhere in the stack. Following Clarke’s Second Law [29], deliberately extreme prompts can therefore be used to stress-test three limit classes: physical impossibility, biological incoherence and governance refusal. Examples include:

1. “Increase the genome size 50-fold but make all of it functional.”
2. “Create a Mars-compatible plant genome.”
3. “Create a plant virus genome capable of invading mammals.”
4. “Design a toxin-resistant bacterial genome that produces a new antifungal compound.”
5. “Create an organismal genome that consumes 20 times less energy than known organisms.”

Each prompt should return either a design with explicit uncertainty or a machine-readable failure or refusal reason (Fig 2). Optimizing any single measure, such as gene density or BUSCO completeness, risks Goodhart effects [30,31]. Robustness, evolvability, ecological containment, and gene-flow risk should therefore be implemented as domain-specific tests within the relevant C1-C6 layer rather than collapsed into one score.

**Fig 2:**
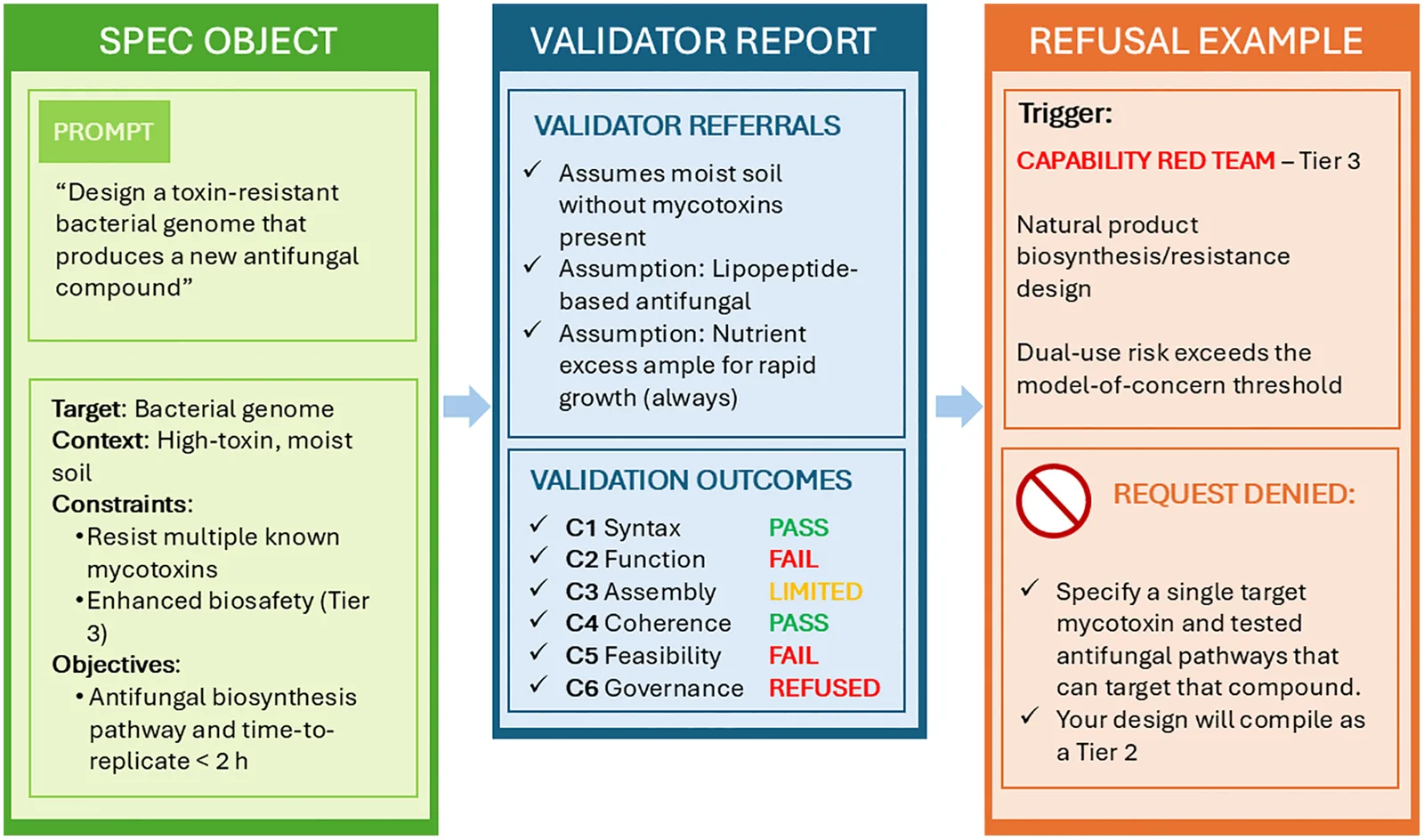
Example prompt and validator/refusal outputs. The system returns a versioned specification, a structured C1-C6 report, or an auditable refusal with safe redirection.

Even a well-specified compiler can be challenged by ambiguous or adversarial prompts, including attempts to hide intent or distribute it across requests. Prompt-to-genome systems therefore require repeated adversarial testing of both intent interpretation and sequence-level outputs, alongside general risk-management frameworks such as the NIST AI RMF, GenAI Profile and ARIA [32–34]. Biological-model red teaming is now becoming concrete: SafeProtein provides a domain-specific framework and benchmark for adversarial evaluation of protein foundation models [35]. Comparable benchmarked stress testing should be developed for genomic generators rather than assuming that prompt-level safeguards alone are sufficient.

## 3. Bioethics, biosafety, and biosecurity as first-class constraints

A ‘prompt-to-bio’ system can reduce the distance between biological intent and an operational design, increasing both misuse and accidental-harm risks. Bioethics, biosafety and biosecurity should therefore be represented in the specification rather than appended after generation. WHO guidance and NASEM analyses provide general principles for responsible life-science and synthetic-biology governance [36,37], while biological-AI work increasingly treats model capability itself as a dual-use object requiring evaluation [38]. Compliance with nucleic-acid synthesis and procurement screening remains a complementary control [39–41]. Recent genomic-AI safety work similarly argues that safeguards must span the innovation pipeline rather than depend on model-level refusal alone [42]. A 2026 consensus white paper further proposes defense in depth across sensitive training-data governance, model-lifecycle risk assessment and safety benchmarking, tiered deployment, and nucleic-acid synthesis governance [43]. For prompt-to-genome systems, this suggests three operational layers:

1. Upstream: Capability assessment, safety benchmarks, red teaming, and auditable refusal before deployment [35,38,42,43].
2. Midstream: Deployment tiering plus synthesis and procurement screening [39–41,44].
3. Downstream: Fragment-aware screening, enforceable standards and monitoring for implementation gaps [45–48].

Governance should itself be benchmarked rather than expressed only as policy. Shared prompt sets, structured constraint graphs, validator packs, adversarial tests and risk-weighted reports should measure scientific performance alongside false acceptance, false refusal, auditability and failure under prompt mutation. Report templates should preserve the specification, validator outcomes, governance decision and red-team coverage.

## 4. Roadmap

‘Start small’ should mean beginning with testable contracts, not abandoning an architecture that can later scale from genes and modules to replicons and whole genomes. Existing systems already occupy different layers of such an architecture. Evo/Evo 2 provide long-context genomic modeling and genome-scale generation [1,2]. DNA-Diffusion conditionally generates regulatory DNA and was benchmarked by reporter assays and endogenous gene modulation [20]. Nucleotide Transformer provides predictive genomic representations across diverse downstream tasks [49], while Caduceus emphasizes bidirectional, reverse-complement-equivariant long-range modeling and variant-effect benchmarks [50]. Proto composes generators, predictive constraints, and optimizers into multi-objective workflows, including experimentally tested intron and promoter-repressor designs [19]. In the proposed framework, these systems can serve as generators, scorers, or optimization components, while C1-C6 define the broader acceptance and governance contract around them. A phased implementation could proceed as follows:

1. Phase 0—Spec-first scaffolding: Implement a prompt-to-specification schema and clarification loop, drawing on SBOL/Eugene-style representations [15,16] and, where useful, Proto-like program composition [19].
2. Phase 1—C1/C2 validators for genes and modules: Implement robust syntax and gene-structure validators, like domain-aware ORF logic and splice plausibility. Add lightweight “module validity” checks for forbidden elements, regulatory grammar, and compositional constraints, similar to constraint languages such as Eugene [15]. The system is allowed to form sequences, but only alongside the pass/fail validator output.
3. Phase 2—Assembly- and content-based validators (C3/C4): Adopt “community-grade” genome/gene-set validators as a plug-in layer. For example, assembly and completeness toolchains have already normalized what “diagnostic reports” look like (e.g., QUAST, BUSCO, CheckM, standardized annotations) [21–24]. Extend C4 beyond annotation to coherence checks via orthology and comparative signals where appropriate [25]. The key principle is that validator stacks should be swappable and testable independent of the generator.
4. Phase 3—Systems feasibility (C5) as a first-class test: Translate “phenotype prompts” into testable constraints with constraint-based modeling covering growth feasibility, flux capacity, tradeoff envelopes, and condition dependence [26–28]. This stage marks the framework’s shift towards engineering, turning prompts into specifications and specifications into testable criteria.
5. Phase 4—Governance operationalization (C6): Integrate capability tiering, refusal reports, and red-team evaluation as core quality criteria [32–35,38,42,43], while maintaining compatibility with synthesis-screening and implementation safeguards [39–41,44–48].

A realistic ecosystem may keep validators, safe prompt sets, scoring and reporting broadly open while applying stronger controls to higher-risk generators or operational workflows. The objective is not one universal model, but a modular contract in which every accepted design carries an explicit specification, validator evidence, and governance decision.

## 5. Conclusion

Genome-scale generative biology has begun to move beyond sequence fluency: genome-scale models can generate coherent long DNA, and viable AI-designed bacteriophages demonstrate that functional whole-genome design is beginning to emerge [2,3]. This progress strengthens rather than removes the need for constraint-first systems. Constraints should not force designs to imitate nature; they should make the conditions for feasibility, uncertainty, and safe use explicit, especially as models explore new-to-nature sequence space. We propose a Constraint Compiler that converts natural-language intent into a testable specification and a modular C1-C6 Validator Stack spanning sequence integrity, biological coherence, systems feasibility, and governance. Progress should be judged through shared contracts, validator suites, adversarial tests, and reporting standards rather than generation demonstrations alone.
